# Potential Biological Control of Schistosomiasis by Fishes in the Lower Senegal River Basin

**DOI:** 10.4269/ajtmh.18-0469

**Published:** 2018-11-21

**Authors:** Martin C. Arostegui, Chelsea L. Wood, Isabel J. Jones, Andrew J. Chamberlin, Nicolas Jouanard, Djibril S. Faye, Armand M. Kuris, Gilles Riveau, Giulio A. De Leo, Susanne H. Sokolow

**Affiliations:** 1 School of Aquatic and Fishery Sciences, University of Washington, Seattle, Washington;; 2 Hopkins Marine Station of Stanford University, Pacific Grove, California;; 3 Biomedical Research Center Espoir Pour La Santé, Saint-Louis, Sénégal;; 4 Santé Plus, Dakar, Sénégal;; 5 Department of Ecology, Evolution and Marine Biology, and Marine Science Institute, University of California, Santa Barbara, California

## Abstract

More than 200 million people in sub-Saharan Africa are infected with schistosome parasites. Transmission of schistosomiasis occurs when people come into contact with larval schistosomes emitted from freshwater snails in the aquatic environment. Thus, controlling snails through augmenting or restoring their natural enemies, such as native predators and competitors, could offer sustainable control for this human disease. Fishes may reduce schistosomiasis transmission directly, by preying on snails or parasites, or indirectly, by competing with snails for food or by reducing availability of macrophyte habitat (i.e., aquatic plants) where snails feed and reproduce. To identify fishes that might serve as native biological control agents for schistosomiasis in the lower Senegal River basin—one of the highest transmission areas for human schistosomiasis globally—we surveyed the freshwater fish that inhabit shallow, nearshore habitats and conducted multivariate analyses with quantitative diet data for each of the fish species encountered. Ten of the 16 fish species we encountered exhibited diets that may result in direct (predation) and/or indirect (food competition and habitat removal) control of snails. Fish abundance was low, suggesting limited effects on schistosomiasis transmission by the contemporary fish community in the lower Senegal River basin in the wild. Here, we highlight some native species—such as tilapia, West African lungfish, and freshwater prawns—that could be aquacultured for local-scale biological control of schistosomiasis transmission.

## INTRODUCTION

The first successful programs to prevent infectious diseases by controlling their nonhuman hosts were carried out at the beginning of the 20^th^ century.^1–4^ More than 100 years later, parasites with complex life cycles continue to affect more than one billion people,^5^ representing one of the gravest ongoing health crises. An exemplary case is schistosomiasis, a neglected tropical disease affecting more than 200 million people in more than 70 countries, primarily in sub-Saharan Africa.^5^ The disease is caused by *Schistosoma* spp. trematodes.^6,7^ Adult schistosomes reside in human (the definitive host) blood vessels surrounding the intestines or bladder and shed eggs that escape the body via urine or feces. If those eggs contact fresh water, they hatch as miracidia that must locate, penetrate, and infect aquatic snails.^8^ The parasite reproduces asexually in its snail host, shedding free-swimming cercariae—as many as 2,000 or more per snail per day^9^—usually for the remaining life of the infected snail. Cercariae infect humans via skin penetration when they walk, bathe, or swim in infested freshwater lakes, ponds, streams, and irrigation canals. Schistosomiasis can cause mild to severe systemic disease, including anemia, growth stunting, chronic pain, fatigue, ascites, diarrhea, impaired cognition, infertility, and organ-specific pathologies, such as urinary dysfunction, kidney disease, enlarged spleen, liver fibrosis, portal hypertension, and increased susceptibility to hepatitis C, human immunodeficiency virus, sexually transmitted diseases, urinary tract infections, and liver and bladder cancers.^10,11^

Control strategies based exclusively on human treatment do not target the transmission of the parasite (i.e., infections in snail hosts).^12,13^ People can be rapidly reinfected after treatment.^14^ Aside from improving access to clean water, hygiene, and sanitation,^15–17^ the greatest successes in schistosomiasis control were historically achieved by integrative measures combining human treatment with interventions targeting snails.^18,19^ Molluscicides have been used extensively in the past and remain cost-effective in some circumstances^20^ but are disadvantaged by their toxicity to nontarget taxa^21^ and the potential for snail recolonization after application.^22^

Biological control—the use of natural enemies to combat pests^23^—can be a targeted and effective strategy for reducing the transmission of human diseases without causing collateral environmental damage (e.g., pollution). This approach ideally uses or augments native species^13,24,25^ to draw down environmental sources of transmission and thereby reduce human risk.^23^ Candidate species are challenging to identify and must be deployed at the effective densities. Non-native species, although potentially effective as biological control agents, might have undesirable nontarget impacts. We, therefore, set out to perform an analysis of native freshwater fauna, in search of candidate species for the control of schistosome transmission stages or their snail intermediate hosts.

Biological control of snail hosts or of parasite free-living stages can be achieved through direct (predation) and indirect (food competition and habitat removal) ecological interactions.^24^ Release of molluscivorous predators has been shown to significantly decrease infections in school children,^13,26^ and predation on free-swimming stages of schistosomes likely occurs in the wild (it is well documented in laboratory studies^27–31^) and, therefore, may also help reduce *Schistosoma* spp. transmission.^32^ Snails forage on detritus, algae, and plants,^24,33–35^ and in addition to being a food source, aquatic macrophytes provide snails with oviposition sites^34^ and shelter from both predation^36^ and wind/wave action.^37^ Thus, taxa competing with snail hosts for the same food resources may reduce disease transmission;^38^ manual removal of aquatic plants is already known to be an effective method in small, enclosed systems.^39^

The goal of our study was to survey the native freshwater fishes (ichthyofauna) of western Senegal, a region that has been plagued by high schistosomiasis burdens since the completion of the Diama Dam in 1986.^14,40^ We sought to identify naturally occurring, potential biological control agents of schistosomiasis that could be cultured at high densities at nearshore sites. We sampled fishes in river and lake littoral habitats—the areas where human activities occur, where infected snails are generally distributed and where most disease transmission presumably takes place^37^—to determine what fish species are present and to assess their relative abundance. We analyzed literature diet data to identify which of the detected fish species may act as direct and/or indirect biological control agents of schistosomiasis.

## METHODS

### Ichthyofaunal composition.

**Figure 1.**
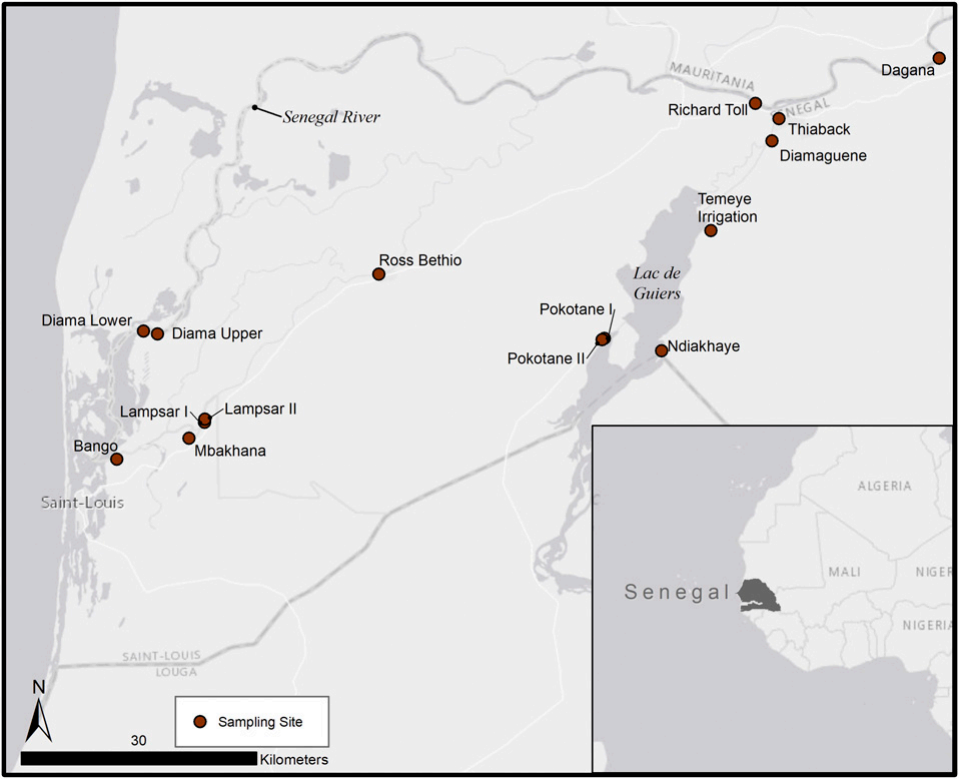
Map of the study region in the lower Senegal River basin. Bodies of (fresh and marine) water are noted in dark grey. International and regional boundaries are indicated by dashed lines. This figure appears in color at www.ajtmh.org.

We sampled ichthyofauna at 15 littoral sites in the lower Senegal River basin (Figure 1) from February 2011 to June 2012 (see Supplemental Appendix Table A1 for sampling time schedule). Sites were selected with input from local epidemiologists and malacologists; they were transmission areas adjacent to villages in a geographical area known to be hyperendemic for schistosomiasis^40^ and included a wide representation of lotic and lentic habitats. Although human epidemiological data at these sites are not available from the study period, a 2009 report by the Ministry of Health and Prevention found that in the ecological region encompassing all of our sites, 42% of school children were infected with intestinal schistosomiasis and 50.2% with urinary schistosomiasis.^41^ In some villages, prevalence reached as high as 88% and 95% for intestinal and urinary schistosomiasis, respectively.^41^ Fish traps (24″ × 12″; Promar, Gardena, CA) were baited with a mixture of fish and plant tissues (*Manihot esculenta* roots, *Tamarix senegalensis* leaves) to attract diverse foraging guilds. We deployed traps during the day and retrieved them approximately 24 hours later to enable capture of both diurnal and nocturnal species. A total of 265 fish traps were deployed for 6,297 trap-hours (Supplemental Appendix Table A1). Most captured fishes were photographed and later identified according to Paugy et al.^42^ Fishes that could not be definitively identified to the genus or species level (either in the field or by photographs) were excluded from our dataset (*N* = 17). All quantitative analyses were conducted in R version 3.3.2.^43^

### Rarefaction.

A rarefaction curve was calculated using the method of Colwell et al.^44^ with the “iNEXT” function and package^45^ to assess whether our samples accurately reflected the full measure of native fish diversity in littoral habitats. To be conservative, genera in which we could not identify any specimens to the species level were each treated as a single species. For genera in which we could only identify a subset of the specimens to the species level, we included only those specimens that were explicitly assigned a species name.

### Dietary niches.

To identify potential biological control agents of schistosomiasis, we surveyed published studies for quantitative data on diet composition of the fish species in our sample and used the available information to: 1) estimate each species’ dietary niche breadth, 2) characterize inter- and intraspecific differences in diet, and 3) estimate relative resource use of each diet item across the populations documented in the literature. Because of varying methods between studies, diet composition data from the literature were divided into two subsets: one by % volume (volume of diet item *X*/volume of all items) and a second by % number (number of diet item *X*/number of all items). Literature sources were quality filtered; those with all diet proportions summing to values more than five percentage points above or below 100% (potentially because of rounding error, miscalculation, or missing data) were excluded. Diet items were aggregated into nine categories: insects, molluscs (including snails), other macroinvertebrates (not including insects or molluscs), zooplankton (potentially including schistosome cercariae), fishes, amphibians, plants, algae, and detritus (organic and inorganic). Unidentified or artificial diet items were excluded. The literature sources we used included diet composition estimates from various countries, habitats, seasons, months, and size classes (Supplemental Appendix Table A2). When a source reported month-specific diet compositions, only a subset of those month-specific datasets were included so as to capture seasonal variability in diet while not over-representing that specific population in the across-population niche breadth and relative resource-use estimates. For genera in which we could not identify specimens to the species level, we used datasets only from species listed as being native within our study region (*Chrysichthys*—*Chrysichthys auratus*, *Chrysichthys nigrodigitatus*; *Clarias*—*Clarias gariepinus*, *Clarias anguillaris*; *Labeo*—*Labeo coubie*) according to Paugy et al.^42^

Principal coordinates (PCs) analyses (PCoAs) of diet composition data by % volume and % number were conducted with the “cmdscale” function from the “stats” package in R^43^ to assess inter- and intraspecific differences in foraging habits. Proportional data were logit-transformed with the “logit” function from the “car” package in R,^46^ with values of 0 replaced by the minimum observed nonzero proportion *P* and values of 1 replaced by 1 − *P*, as recommended by Warton and Hui.^47^ The Euclidean distance metric was used in both ordinations. Principal coordinate loadings were calculated using 5,000 permutations with the “envfit” function from the “vegan” package in R.^48^

The overall dietary niche breadth of each species of fish and relative resource use of each of the nine diet categories were calculated with the method of De Cáceres et al.,^49^ which in this case provides bootstrap estimates, across populations, informed by both the untransformed % volume and % number diet composition datasets. Volumetric and numeric datasets (from different sources) were both included (when available) to provide a measure of resource use balancing these composition metrics because diet items that constitute a large volume may be numerically rare and those that constitute a small volume may be numerically abundant.^50^ For example, the single estimate of relative resource use by *Hemichromis fasciatus* across the available studies was determined by bootstrapping two % volume and three % number datasets, whereas that for *Clarias* spp. was derived from five % volume and four % number datasets (Table 2 and Supplemental Appendix Table A2). In instances where a single literature source provided diet composition for the same species/population in both % volume and % number (e.g., *Synodontis ocellifer*^51^), only the % volume data were included to avoid pseudoreplication. Relative resource use is reported here as a percent but not more specifically as % volume or % number because, depending on the species, the bootstrapped estimate may be derived from only volume, only number, or both volume and number datasets. Dietary niche breadth and relative resource use of each diet category were calculated with the “nichevar” and “nichepref” functions from the “indicspecies” package in R.^52^

### Water chemistry.

The similarity of sampling sites with regard to water chemistry—and thus their suitability as potential control agent stocking sites—was assessed with PCoA. Water chemistry data were collected at each site at the time of fish collection and, when possible, included pH, temperature (°C), salinity, ammonium (mg/L NH_4_), nitrate (mg/L NO_3_), nitrite (mg/L NO_2_), phosphate (mg/L PO_4_), calcium hardness (mg/L Ca), alkalinity (mg/L CaCO_3_), magnesium (mg/L Mg), and iron (mg/L Fe). Data were collected with a YSI pH10 pen, YSI 9300 photometer (YSI, Yellow Springs, OH), and salinity refractometer. Values for each metric were averaged across visits and mean-standardized. Gower’s coefficient^53^ was used to calculate the distance matrix.

## RESULTS

### Ichthyofaunal composition.

We detected 16 fish species from 13 genera in 13 families (Table 1). The dominant taxa were *Synodontis*, *Polypterus*, and *Hemichromis* species (particularly *Synodontis schall*, *Polypterus senegalus*, and *Hemichromis bimaculatus*), which together constituted 86.6% of the total number of specimens (*N* = 366) caught across all sites. Although the presence of *Paradistichodus dimidiatus* in the Senegal River was first reported by Dorfman and Sagna,^54^ we believe our collection of one specimen near the town of Ndombo in a canal connecting Lac de Guiers to the lower Senegal River (16°25′56.71″N, 15°42′7.24″W) represents the downriver-most record. No exotic species were encountered.

**Table 1:** Overall composition of fish (ichthyofauna) captured at all sampling sites

Family	Species	*N*	Relative abundance
Anabantidae	*Ctenopoma petherici*	1	0.3%
Channidae	*Parachanna obscura*	9	2.5%
Cichlidae	*Hemichromis bimaculatus*	29	7.9%
	*Hemichromis fasciatus*	1	0.3%
Citharinidae	*Citharinus citharus*	1	0.3%
Clariidae	*Clarias* spp.	2	0.5%
Claroteidae	*Chrysichthys* spp.	11	3.0%
Cyprinidae	*Labeo* spp.	2	0.5%
Distichodontidae	*Paradistichodus dimidiatus*	1	0.3%
Malapteruridae	*Malapterurus electricus*	3	0.8%
Mochokidae	*Synodontis nigrita*	10	2.7%
	*Synodontis ocellifer*	9	2.5%
	*Synodontis schall*	108	29.5%
	*Synodontis* spp.	66	18.0%
Polypteridae	*Polypterus senegalus*	78	21.3%
	*Polypterus* spp.	16	4.4%
Protopteridae	*Protopterus annectens*	4	1.1%
Schilbeidae	*Schilbe intermedius*	15	4.1%

**Figure 2.**
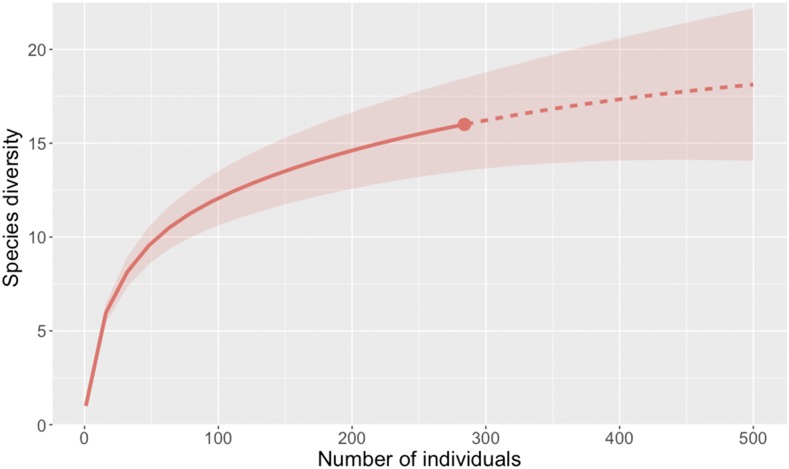
Rarefaction curve of observed fish species. Interpolation of observed species (solid line), extrapolation to 500 individuals (dashed line), and 95% confidence interval (faded red) are included. Genera in which we could not identify any specimens to the species level (*Chrysichthys*, *Clarias*, and *Labeo*) were each treated as a single species. For genera in which we could not identify a subset of the specimens to the species level (*Polypterus*, *Synodontis*), we included only those specimens that were explicitly assigned a species name. Thus, the sample size reflected in this figure is less than that presented in Table 1. This figure appears in color at www.ajtmh.org.

Although Paugy et al.^42^ list the taxonomically confounded *Ctenopoma petherici* and *Ctenopoma kingsleyae* as present within our study region, we identified our lone specimen with the meristics-based canonical variate scoring equation of Norris and Douglas,^55^ which unambiguously assigned it as *C. petherici*. The *Chrysichthys*, *Clarias*, *Labeo*, and a subset of the *Synodontis* and *Polypterus* specimens could not be identified to the species level because of poor specimen and/or photograph quality. Rarefaction indicated that we sampled a large proportion but not all of the species present in littoral habitats of our study region, as evinced by the rarefaction curve approaching an asymptote (Figure 2). Additional non-fish organisms captured in the traps included crabs (*Callinectes* spp.), prawns (*Atya* spp., *Caridina* spp. *Macrobrachium vollenhovenii*), and turtles (*Pelusios* spp.).

### Dietary niches.

Literature surveys of quantitative diet composition data for our sampled fish species yielded 30 datasets of diet composition from 14 bibliographic sources by % volume and 29 datasets from 11 sources by % number. We obtained data for all of our sampled genera and species except *P. dimidiatus* (Supplemental Appendix Table A2), which is excluded from the following results.

Five diet categories are relevant for schistosomiasis control: molluscs, zooplankton, plants, detritus, and algae. Note that none of the literature diet datasets explicitly mentioned trematode cercariae as a stomach content. Kaplan et al.^56^ showed that for cercariae, fish predation must be documented within minutes of capture because these small soft-bodied zooplankters are very rapidly digested. Zooplanktivory, therefore, indicates the potential for consumption of cercariae.^57^ Ten of 16 species in our sample include one or more of the control categories in their diet for ≥ 10% of their relative resource use (Table 2). The sole substantial consumer of molluscs was *Protopterus annectens*. The only species with high relative resource use of zooplankton, *Labeo* spp., had a value of 15%, meaning minimal consumption of cercariae at best. Similarly, only *Labeo* spp. exhibited a relative resource use of plants ≥ 10%. Nine species exhibited notable detritivory, including five with a relative resource use ≥ 30% for detritus. Among the four more substantially algivorous species, *Labeo* spp. and *Citharinus citharus* exhibited a relative resource use > 30% for algae. Of the 10 potential control species, two might exert both direct and indirect effects on schistosomiasis transmission through their feeding preferences (i.e., consumption of molluscs/zooplankton and plants/algae/detritus), whereas the other eight might only exert indirect effects (i.e., consumption of plants/algae/detritus but not molluscs/zooplankton).

**Table 2:** Fish species with an estimated relative resource use (proportion of the diet) ≥ 10% for at least one of the diet categories that may result in direct or indirect control of schistosomiasis

		Direct		Indirect		
	Species	Molluscs	Zooplankton	Plants	Detritus	Algae
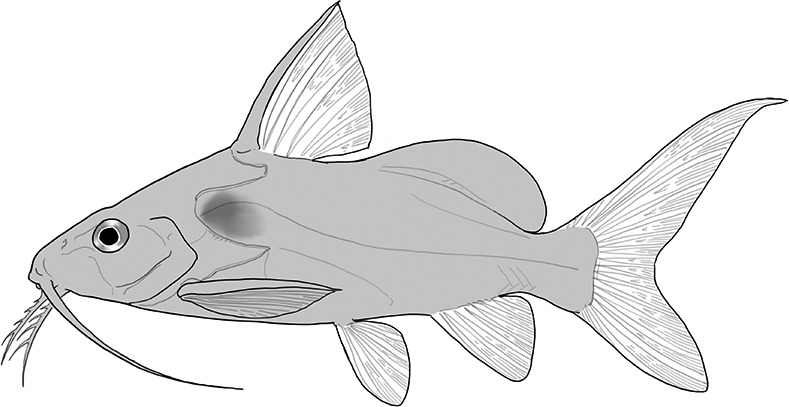	*Synodontis schall*	–	–	–	22	11
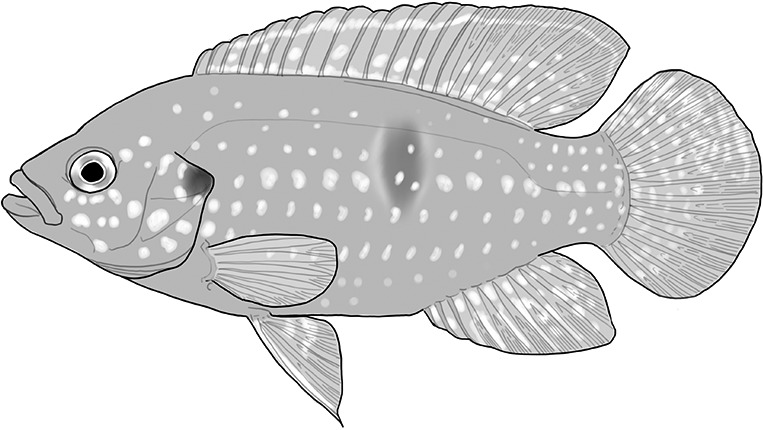	*Hemichromis bimaculatus*	–	–	–	–	15
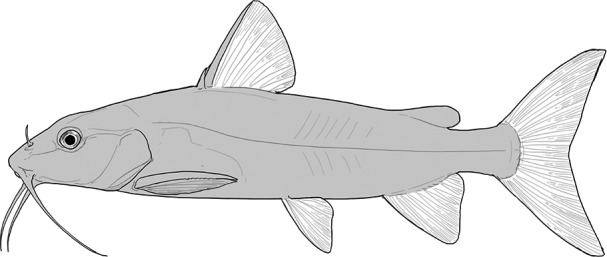	*Chrysichthys* spp.	–	–	–	30	–
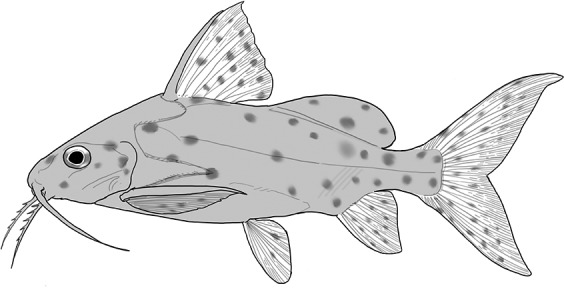	*Synodontis nigrita*	–	–	–	15	–
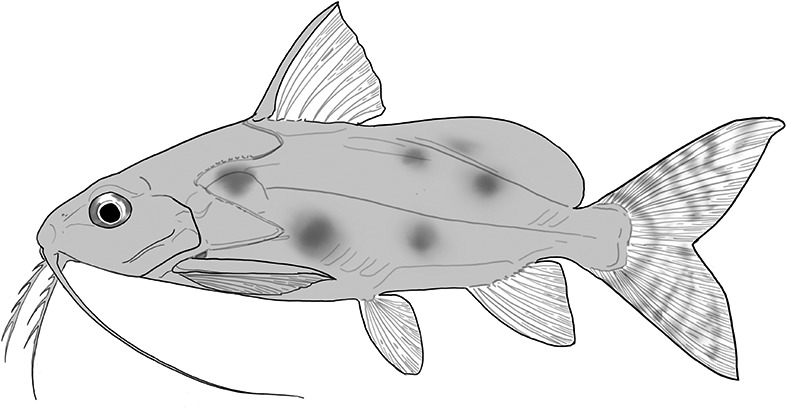	*Synodontis ocellifer*	–	–	–	94	–
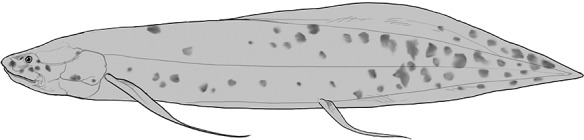	*Protopterus annectens*	50	–	–	48	–
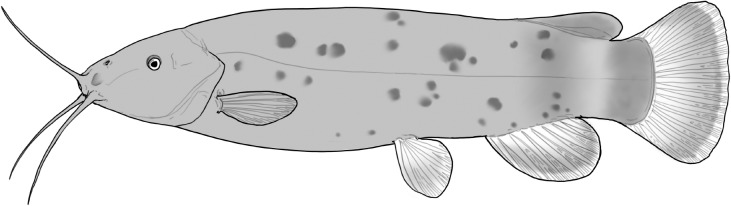	*Malapterurus electricus*	–	–	–	10	–
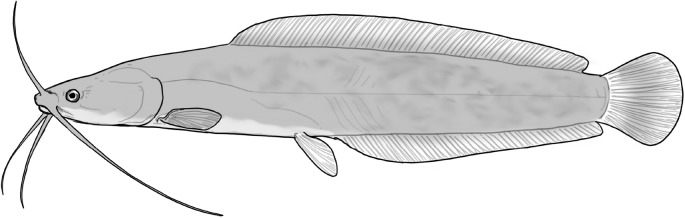	*Clarias* spp.	–	–	–	11	–
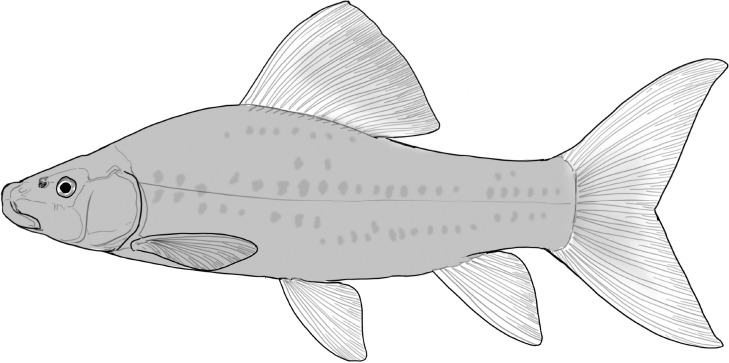	*Labeo* spp.	–	15	19	33	33
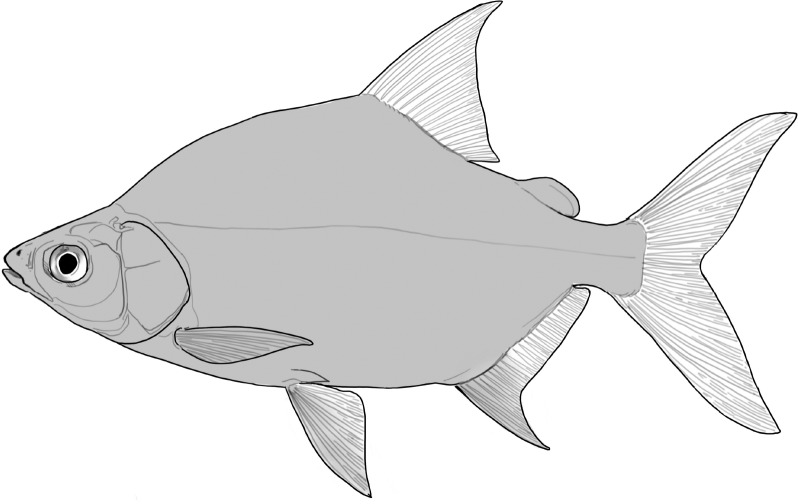	*Citharinus citharus*	–	–	–	43	57

Relative resource use is estimated by bootstrapping both volumetric and numerical diet composition datasets (see Methods: *Dietary Niches*) and is reported here to the nearest whole percent (values < 10% are not included). Species are listed in order of decreasing relative abundance. (Artwork copyright of Brandon Li.).

**Figure 3.**
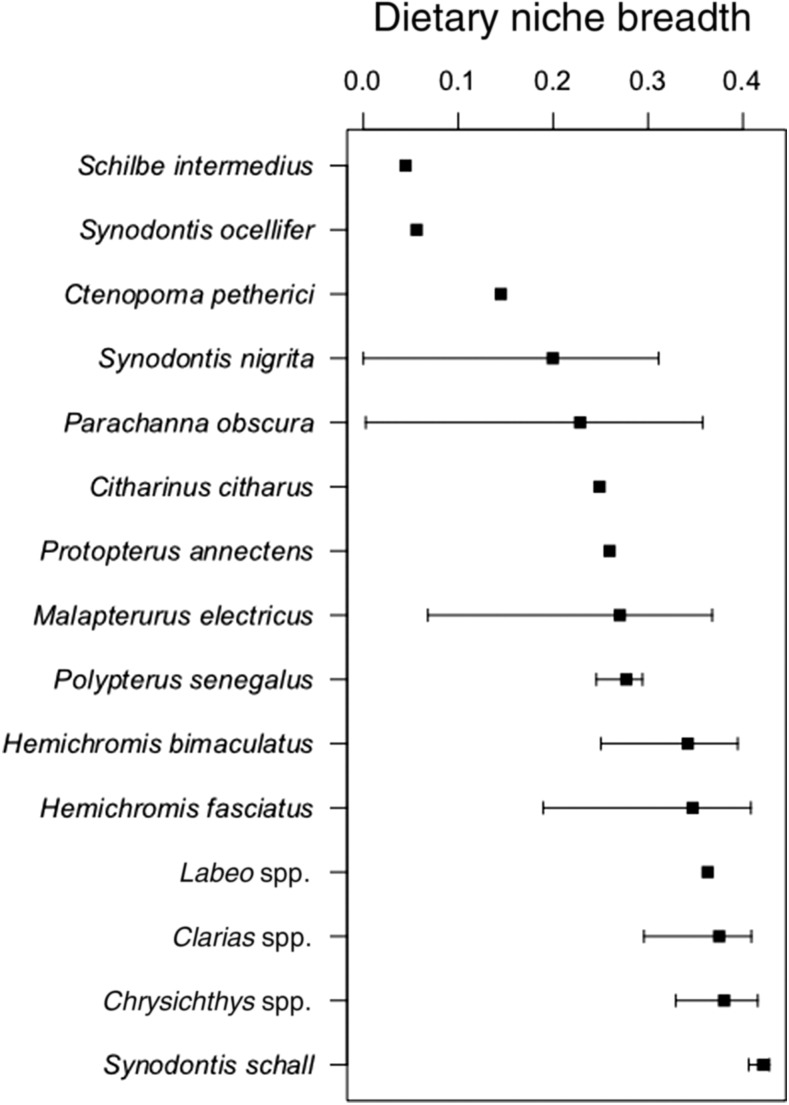
Dietary niche breadth estimated from the available diet composition literature of each species/genus observed in this study. Species/genera are ordered from least (top) to greatest (bottom) dietary niche breadth. Error bars represent 95% confidence intervals. Species estimated from less than three datasets do not have error bars. Genera level estimates are calculated from datasets for native species of that genus (see Methods: *Dietary Niches*).

Estimates of dietary niche breadth ranged from 0.0445 (*Schilbe intermedius*) to 0.4213 (*S. schall*), representing a gradient from specialist to generalist foraging behavior (Figure 3). Species with wide confidence intervals represented divergence in dietary breadth among populations represented in the literature. For example, *Malapterurus electricus* consumed all nine diet categories in one study^58^ and only four in another.^59^ By contrast, species with tighter confidence intervals, such as *S. schall*, demonstrated consistent levels of niche breadth among populations.^51,60–62^ Potential control species (Table 2) spanned nearly the full observed range of niche breadth, from influencing only one direct or indirect control category (e.g., *S. ocellifer*) to most or all of the direct and indirect control categories (e.g., *Labeo* spp.).

In the % volume ordination, most of the diet composition variation between and within species was explained by differences in the consumption of items related to indirect control of schistosomiasis (i.e., consumption of detritus, algae) and those not pertaining to direct or indirect control (i.e., consumption of fishes, insects). Principal coordinate 1 (54.1%) loaded fishes (0.926), plants (0.890), and zooplankton (0.036) against all other categories (–0.170 to –0.825). Principal coordinate 2 (29.1%) loaded insects (–0.707), other macroinvertebrates (–0.565), plants (–0.456), and fishes (–0.377) against the remaining categories (0.944–0.999).

In the % number ordination, inter- and intraspecific variation in diet composition was largely explained by the same diet items as the % volume ordination (detritus, algae, fishes, and insects) but also included zooplankton as a significant category. Principal coordinate 1 (44.8%) loaded fishes (–0.985) against all other categories (0.374–0.993) except amphibians (0). Principal coordinate 2 (30.4%) loaded zooplankton (–0.928), algae (–0.918), detritus (–0.894), plants (–0.554), and other macroinvertebrates (–0.505) against the remaining categories (0.115–0.882) except amphibians (0). The inclusion of zooplankton as a significant diet item in the % number PCoA but not the % volume PCoA was probably attributable to differences in other diet categories between dataset types (number versus volume), as it was not attributable to a higher variance or average % composition value of zooplankton in the % number datasets than the % volume datasets (one-tailed *F*-test: *F*_28,29_ = 1.16, *P* = 0.35; one-tailed *t*-test: *t*_57_ = 0.35, *P* = 0.64), as might be expected for numerically abundant but volumetrically minute organisms.

### Water chemistry.

Sampling sites were largely similar with respect to water chemistry, with the exceptions of Temeye and Diama Lower (Supplemental Figure 1). The Temeye irrigation drainage at the northeast end of Lac de Guiers exhibited the highest nitrate and nitrite, and comparatively high hardness, alkalinity, and iron, possibly the result of pollution documented in this area of the lake.^63^ Diama Lower, located just below the Diama Dam, exhibited the highest salinity, calcium hardness, ammonia, and magnesium. The different water chemistry below the dam, most notably increased salinity, is due to saltwater penetration, which is prevented above the dam.^64^

## DISCUSSION

Our sampling of the native ichthyofauna, in concert with analyses of corresponding literature diet data, suggest several native fish as potential biological control agents of schistosomiasis in the lower Senegal River basin. Although 62.5% of fish species we captured may serve as natural enemies of snails, most of the identified pathways for control were indirect (i.e., via consumption of algae and detritus, on which snails feed, and plants, on which snails feed, seek shelter, and reproduce) rather than direct (i.e., predation on snails). Although we observed low relative abundance of species with the highest estimated degree of snail foraging, some of these species could potentially be cultured and stocked to reduce local schistosomiasis transmission.

The low abundance of species with potential for disease control may explain why the lower Senegal River basin has one of the highest schistosomiasis transmission rates in the world. For example, in contrast to multiple snail-eating fish species directly controlling snail populations in Lake Malawi,^65,66^ we found only one substantially molluscivorous species, the West African lungfish (*P. annectens*),^67^ in our samples. It occurs at such low densities that it probably exerts only minimal control over snail populations in the study region. Although the West African lungfish has been observed to consume freshwater prawns,^68^ a potential conflict for snail control efforts, it is probably still the best choice for aquaculture or restoration because individuals can consume hundreds of snails per day in experimental settings, prefer to eat snails even when presented with multiple prey alternatives,^69^ and are tolerant of low oxygen conditions^70^ that may occur in areas of limited water flow. In addition to molluscivores, restoration or aquaculture to augment species of herbivorous fishes, such as *Labeo* species,^71–73^ could help eliminate resources vital to snails and thereby curb the transmission of schistosomiasis.^24,38^

Fishes that might compete with snails for food and/or destroy their plant habitat are present in the lower Senegal River basin, but their diet variability and low relative abundances probably limit their effectiveness in combating schistosomiasis in the wild today. For example, African jewel cichlids (*H. bimaculatus*) were moderately common (7.9% relative abundance, Table 1) at our sampling sites but exhibit variable dietary composition and niche breadth depending on the population. This species was almost exclusively piscivorous^74^ or insectivorous^67^ in some areas but exhibited a more generalized diet largely dependent on algae in another area.^75^ With the exception of *S. schall*, the remaining species with potential for indirect control all had relative abundances ≤ 3%, suggesting minimal snail or schistosome control across the contemporary landscape of the lower Senegal River basin.

One of the upside-down catfishes, *S. schall*, might be the only species in the lower Senegal River basin with the dietary habits and sufficient contemporary abundance to naturally reduce transmission of schistosomiasis. This species consumes all five diet categories that might contribute to snail control, including snails themselves,^21,76^ but as part of a broad diet in which only detritus and algae typically constitute > 10% of the relative resource use (Table 2). The foraging mode of *Synodontis* species is highly flexible, as they are known to bottom-feed, filter-feed, defoliate macrophytes, consume fish scales, and invert themselves dorsoventrally to surface-feed,^61,76–78^ although inversion is uncommon for *S. schall*.^79^ *Synodontis schall* is the most abundant species in the area, consistent with its status as the most common mochokid catfish in other ecosystems.^51,80^ However, the remarkable niche breadth of *S. schall*^51,60–62^ diminishes its use for targeted biological control, as it may consume nontarget taxa, including other biological control species such as fish and prawns.

A number of fish species previously reported from the lower Senegal River basin^42^ were notably absent from our collections. For example, we encountered none of the tilapia that are reportedly native to the region (species of *Tilapia*, *Oreochromis*, and *Sarotherodon*), even though surveys in the early and late 1990s, using different sampling gear, found some to be particularly abundant.^81,82^ *Tilapia zilli* consume macrophytes and exhibit dietary flexibility to consume detritus^83^ and snails^84^ as aquatic plants become scarcer. Similarly, *Tilapia guineensis*, *Oreochromis niloticus*, *Oreochromis aureus*, *Sarotherodon melanotheron*, and *Sarotherodon galileus* primarily forage on macrophytes, detritus, and/or algae.^67,75,85,86^ Although rarefaction indicated that more intensive sampling would have yielded more species, it may be the case that certain native species (including tilapias) were not sampled because of trap design/location or contemporary scarcity caused by overfishing, pollution, and/or environmental changes after the construction of the Diama Dam in 1986.^81,87,88^ For example, limited water release restricts fishes from foraging or spawning in downstream floodplains,^89^ increases salinity experienced by fishes downstream,^87^ and the dam blocks the upriver migration of native, snail-eating river prawns (*M. vollenhovenii*).^90,91^

Although fish abundance in the Senegal River has declined from the pre-dam period,^81^ some of the native species, including Nile tilapia (*O. niloticus*), are already domesticated for aquaculture^92^ and could be cultured to reduce schistosomiasis transmission. We envision that fishes and other natural enemies of snails could be reared in small-scale aquaculture facilities, being repeatedly stocked into netted enclosures at transmission sites along the rivers, lakes, and canals in the region, thereby providing augmentative biological control. Because snails hidden in macrophytes may evade predation,^93^ aquaculture of herbivores such as tilapia (which remove snail shelter) in concert with a benthic molluscivore such as the West African lungfish or *Macrobrachium* river prawns (which can then have greater access to snails) may increase the effectiveness of nearshore snail-control enclosures. In Bangladesh, mixed culture of common carp (*Cyprinus carpio*) and Nile tilapia reduced mollusc abundance and weed biomass in experimental rice fields.^94^ The feasibility of stocking potential biological control taxa at transmission sites is dependent on matching the physiological tolerance and pollution resistance of the stocked species with local environmental conditions and water quality. Sites with extreme conditions or pervasive pollution are unlikely to be suitable for many taxa. Thus, species with broad tolerance and high resistance constitute ideal candidates for biocontrol programs.

We identified potential biological control agents of schistosomiasis based on literature diet data. However, confirmation of a species as a natural enemy of a particular snail species or a predator of cercariae requires quantitative diet analysis in the location of interest; foraging habits of a given species may change depending on the niches occupied by other species in sympatry^95^ and the relative abundance and energetic profitability of the available prey items.^93^ Our 6,000+ hours of trap deployment successfully captured many species and provided valuable data on their relative abundance; however, a more exhaustive ichthyofaunal survey could be achieved by using multiple sampling gear types, so as to avoid sampling bias, at an expanded number of sites. With data of finer temporal and spatial resolution, the relationship between fish abundance and diversity and snail abundance could be investigated. In addition, a survey of fish landings by artisanal fishers could help quantify harvest of species with value for biological control.

Overfishing of some fish species may relax biological control of snails and their habitats and could result in elevated transmission of schistosomiasis.^96^ Elsewhere in Africa, such as at Lake Malawi, fishing bans have been recommended for littoral zones adjacent to human settlements to increase population densities of molluscivorous fishes and thereby reduce that of intermediate host snails.^66^ However, the importance of fish protein to the diet of people in western Senegal—and indeed much of Africa—makes this untenable. Instead, polyculture of select native fish species in nearshore enclosures may improve human health while simultaneously providing income and food to local communities.^13,97^ Future studies in the Senegal River basin should assess harvest pressure on the native fish communities to determine if any biological control species are at risk and test the effectiveness of mixed fish species and fish/*Macrobrachium* polyculture enclosures to evaluate which combinations may best curtail schistosomiasis transmission.

## Supplementary Material

Supplemental figure and appendix tables
