# Supplementary material for: Potential Biological Control of Schistosomiasis by Fishes in the Lower Senegal River Basin
Source: Am J Trop Med Hyg. 2018 Nov 21;100(1):117–26. doi: 10.4269/ajtmh.18-0469 (PMC6335894; doi:10.4269/ajtmh.18-0469)
Supplement: Supplementary file 1 [file tpmd180469.SD1.pdf]

Supplemental Appendix

Table A1 - Time schedule of sampling at the 15 littoral sites in the lower Senegal River basin. The numbers below the month abbreviations indicate the number of fish traps set in that month. Asterisks (*) indicate the subset of sites sampled in a given month.

	2011											2012					
	F	M	A	M	J	J	A	S	O	N	D	J	F	M	A	M	J
	22	0	22	0	18	4	22	0	17	12	36	0	28	28	28	24	4
Thiabakh	*		*		*		*			*	*						
Temeye	*		*		*		*			*	*						
Ross Bethio	*		*			*	*		*		*						
Richard Toll	*		*		*		*			*	*						
Pokotane I									*		*		*	*	*	*	
Pokotane II									*				*	*	*	*	
Ndiakhaye	*		*		*		*			*	*						
Mbakhana	*		*			*	*		*		*		*	*	*	*	
Lampsar I	*		*		*		*		*		*		*	*	*	*	
Lampsar II									*		*		*	*	*	*	
Diameguene										*	*		*	*	*	*	

Diama Upper	*	*	*	*	*		*	*	*	*	*
Diama Lower	*	*	*	*	*		*				
Dagana	*	*	*	*		*	*				
Bango	*	*	*	*	*		*				

Table A2 – Literature sources used in the quantitative diet analyses. Each study's data type (% volume or number), country, and habitat are included. Studies that divided their datasets by season, month, or size class are indicated.

Species	Data Type	Source	Country	Habitat	Factor
Chrysichthys auratus	Volume	67	Benin	lagoon	
Chrysichthys auratus	Volume	98	Benin	river	season
Chrysichthys auratus	Volume	98	Benin	river	season
Chrysichthys nigrodigitatus	Volume	67	Benin	lake	
Chrysichthys nigrodigitatus	Volume	67	Benin	lagoon	
Chrysichthys nigrodigitatus	Volume	99	Benin	lake	
Chrysichthys nigrodigitatus	Number	100	Nigeria	river	
Chrysichthys nigrodigitatus	Number	101	Nigeria	river	
Citharinus citharus	Volume	102	Nigeria	lake	
Citharinus citharus	Number	60	Sudan	lake	

Clarias anguillaris	Volume	103	Nigeria	floodplain	month
Clarias anguillaris	Volume	103	Nigeria	floodplain	month
Clarias gariepinus	Volume	104	Zambia	floodplain	
Clarias gariepinus	Volume	105	Ethiopia	lake	size class
Clarias gariepinus	Volume	105	Ethiopia	lake	size class
Clarias gariepinus	Number	60	Sudan	lake	
Clarias gariepinus	Number	60	Sudan	floodplain	
Clarias gariepinus	Number	106	South Africa	lake	season
Clarias gariepinus	Number	101	Nigeria	river	
Ctenopoma petherici	Number	60	Sudan	floodplain	
Hemichromis bimaculatus	Volume	67	Benin	lagoon	
Hemichromis bimaculatus	Volume	107	Nigeria	stream	month
Hemichromis bimaculatus	Volume	107	Nigeria	stream	month
Hemichromis bimaculatus	Volume	107	Nigeria	stream	month
Hemichromis bimaculatus	Number	74	Nigeria	river	
Hemichromis bimaculatus	Number	75	Nigeria	lake	
Hemichromis bimaculatus	Number	101	Nigeria	river	
Hemichromis fasciatus	Volume	67	Benin	lagoon	
Hemichromis fasciatus	Volume	108	Ghana	lake	
Hemichromis fasciatus	Number	74	Nigeria	river	
Hemichromis fasciatus	Number	60	Sudan	lake	

Hemichromis fasciatus	Number	101	Nigeria	river	
Labeo coubie	Number	72	Nigeria	river	
Malapterurus electricus	Volume	58	Nigeria	lagoon	
Malapterurus electricus	Number	59	Nigeria	river	
Malapterurus electricus	Number	59	Nigeria	lake	
Parachanna obscura	Volume	67	Benin	lagoon	
Parachanna obscura	Number	74	Nigeria	river	
Parachanna obscura	Number	60	Sudan	lake	
Parachanna obscura	Number	101	Nigeria	river	
Polypterus senegalus	Volume	67	Benin	lagoon	
Polypterus senegalus	Number	74	Nigeria	river	
Polypterus senegalus	Number	60	Sudan	lake	
Polypterus senegalus	Number	60	Sudan	floodplain	
Polypterus senegalus	Number	109	Nigeria	lake	
Protopterus annectens	Volume	67	Benin	lagoon	
Schilbe intermedius	Volume	104	Zambia	floodplain	
Synodontis nigrita	Volume	78	Nigeria	lake	size class
Synodontis nigrita	Volume	78	Nigeria	lake	size class
Synodontis nigrita	Volume	51	Nigeria	river	
Synodontis nigrita	Number	78	Nigeria	lake	size class

Synodontis nigrita	Number	78	Nigeria	lake	size class
Synodontis nigrita	Number	51	Nigeria	river	
Synodontis ocellifer	Volume	51	Nigeria	river	
Synodontis ocellifer	Number	51	Nigeria	river	
Synodontis schall	Volume	61	Ethiopia	lake	
Synodontis schall	Volume	62	Nigeria	river	
Synodontis schall	Volume	51	Nigeria	river	
Synodontis schall	Number	60	Sudan	lake	

Table A3 – Fish species by site. Species presence (x) and absence at the 15 littoral sites sampled from 2011-2012. Note that some specimens, including the lone *Citharinus citharus*, were missing

spatial location data (c.a. 90% of the identified specimens had spatial reference data that could link them to a particular site, while 10% were missing spatial coordinate data).

Species	Bango	Dagana	Diama Lower	Diama Upper	Diameguene	Lampsar I	Lampsar II	Mbakhana	Ndiakhaye	Pokotane I	Pokotane II	Richard Toll	Ross Bethio	Temeye	Thiabakh
Chrysichthys spp.	x		x			x	x	x					x		
Clarias spp.						x									
Ctenopoma petherici													x		
Hemichromis bimaculatus						x	x	x	x				x	x	x
Hemichromis fasciatus													x		
Labeo spp.								x	x						
Malapterurus electricus		x		x				x							
Parachanna obscura		x		x	x	x		x							
Paradistichodus dimidiatus					x										
Polypterus senegalus				x	x	x	x		x	x	x	x	x	x	
Polypterus spp.		x		x				x	x			x		x	
Protopterus annectens				x	x									x	
Schilbe intermedius	x			x		x									x
Synodontis nigrita							x					x			x
Synodontis ocellifer		x		x	x	x		x				x			
Synodontis schall	x	x		x	x	x	x					x	x		x
Synodontis spp.	x	x		x	x	x		x				x		x	x

Figure S1 – Ordination of sampling sites by water chemistry. Percent variation explained by PC 1 and PC 2 are included in the axis labels. The Temeye and Diama Lower sites are labeled to highlight their dissimilarity from the other sites.
