# Supplementary figures and images for: Potential Biological Control of Schistosomiasis by Fishes in the Lower Senegal River Basin
Source: Am J Trop Med Hyg. 2018 Nov 21;100(1):117–26. doi: 10.4269/ajtmh.18-0469 (PMC6335894; doi:10.4269/ajtmh.18-0469)

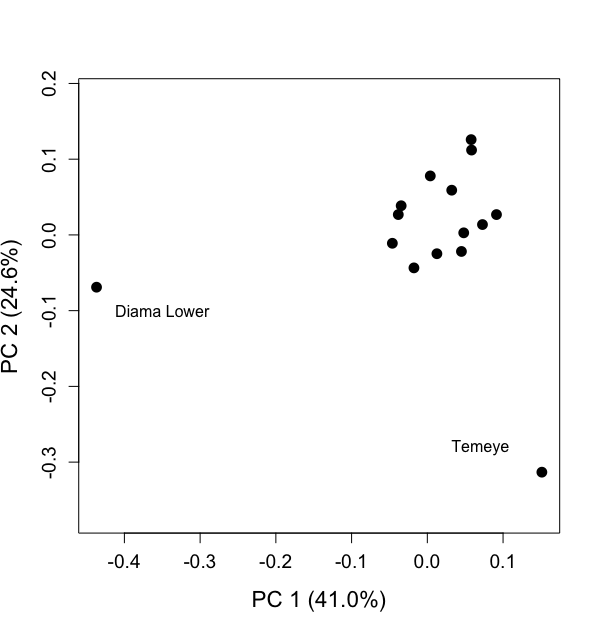

Supplement: Supplementary file 2 [file tpmd180469.SD2.tiff]
